# A structure-aware and causal-invariant framework for glioma cell classification in pathological images

**DOI:** 10.3389/fcell.2026.1861800

**Published:** 2026-06-12

**Authors:** Taishan Chen, Ying Zhang, Cai Jing, Yao Liu, Tanjun Wei, Shujun Xia, Xianhai Li

**Affiliations:** 1 Department of Neurosurgery, Dazhou Central Hospital, Dazhou, China; 2 Department of Pharmacy, Dazhou Integrated TCM & Western Medical Hospital, Dazhou, China

**Keywords:** causal-invariant discrimination, glioma cell classification, morphological relation modeling, pathological image analysis, transformer

## Abstract

**Introduction:**

Accurate classification of glioma cells and normal cells remains challenging due to insufficient fine-grained pathological feature representation, limited modeling of morphological relationships, and interference from staining variation, background noise, and imaging bias.

**Methods:**

This study proposes a hybrid classification framework that integrates a convolutional residual local detail enhancement front-end, a Transformer backbone, a morphological relation modeling mechanism, and a causal-invariant discrimination module. The framework enhances nuclear boundaries, staining textures, and local heterogeneity, models cross-regional contextual and morphological correlations, and suppresses non-essential disturbance factors.

**Results:**

Experiments on a dataset of 473 subjects and 859 pathological images show that the proposed method achieves 98.46% accuracy, 97.94% precision, 98.07% recall, and 0.987 AUC, outperforming competing methods. Ablation and visualization results further confirm the effectiveness of the key modules.

**Discussion:**

The proposed method improves fine-grained feature representation, structural relation modeling, and classification robustness, providing reliable technical support for intelligent glioma-related pathological cell recognition.

## 1 Introduction

Glioma is one of the most common and highly heterogeneous primary tumors in the central nervous system. It is characterized by complex cellular morphology, diverse tissue structures, and is often accompanied by blurred boundaries, obvious staining variations, and highly uneven local microenvironments, thereby imposing high demands on the accuracy, stability, and consistency of pathological diagnosis. With the continuous development of digital pathology and medical artificial intelligence, deep learning-based medical image analysis methods have shown substantial potential in pathological image recognition, subtype discrimination, and computer-aided diagnosis, providing a new technical pathway for the automated understanding of complex pathological phenotypes Li et al. (2023). Meanwhile, the systematic application of deep learning in digital pathology has continued to expand. Relevant studies have shown that pathological image analysis is gradually shifting from traditional handcrafted features and shallow classification methods toward intelligent analysis frameworks capable of jointly modeling local textures, spatial context, and high-level semantic information Janowczyk and Madabhushi (2016). For the glioma cell versus normal cell classification task, how to accurately extract stable discriminative features with pathological significance from complex microscopic visual patterns is related not only to fine-grained recognition capability at the cytological level, but also to the further practical deployment and promotion of intelligent pathology-assisted diagnosis technologies.

In recent years, extensive studies have been conducted worldwide on glioma pathological image analysis and intelligent recognition in digital pathology. Early work demonstrated that deep convolutional networks could be applied to grading and classification tasks of glioma pathological images, and preliminarily verified the feasibility of automated analysis in digital pathology scenarios Ertosun and Rubin (2015); Yonekura et al. (2018). Subsequently, this research direction was further extended from perspectives such as joint modeling of glioma classification and grading, molecular information fusion, and direct prediction of brain tumor subtypes from pathological images Pei et al. (2021); Hewitt et al. (2023). In addition, weakly supervised learning, transfer learning, systematic reviews, and methodological summaries for glioma histopathological classification have continuously promoted the development of this field Zuo et al. (2025); Despotovic et al. (2024); Sun et al. (2023); Redlich et al. (2024); Kurc et al. (2020). In the broader context of intelligent pathological image analysis, methods such as whole slide image classification, self-learning sampling strategies, fine-grained visual-semantic interaction, and vision Transformers based on sequential learning have continuously emerged, further improving pathological image representation learning and global discriminative modeling capability Sali et al. (2020); Fu et al. (2024); Li et al. (2024); Lee and Kwak (2023).

Although existing studies have achieved significant progress in pathological image classification, several key challenges still remain for the fine-grained classification task of glioma cells and normal cells. First, discriminative cues in pathological cell images are often distributed simultaneously in nuclear boundaries, staining textures, local heterogeneity, and inter-regional structural relationships. Therefore, relying solely on convolutional networks or global attention mechanisms may still make it difficult to simultaneously account for fine-grained cytological features and cross-regional morphological organizational patterns. Second, with the increasing application of Vision Transformers and convolution-integrated frameworks in pathological image classification, how to preserve local pathological details while enhancing global modeling capability remains an issue that deserves continuous attention Rahman (2025). Third, real pathological images often contain non-essential disturbance factors such as staining variation, background noise, differences in slice quality, and imaging bias. Although foundation models, self-supervised learning, and representation learning studies in medical imaging have provided important insights for cross-scenario generalization, how to further implement these ideas in stable discriminative modeling for cell-level classification tasks still requires in-depth exploration Xu et al. (2024); Claudio Quiros et al. (2024); Huang et al. (2023); Tsai et al. (2023). Therefore, constructing a classification framework that can simultaneously strengthen local detail representation, explicitly model cellular morphological relationships, and suppress the influence of non-critical disturbances has clear theoretical significance and practical value.

To address the above issues, this study develops a hybrid classification framework for the glioma cell versus normal cell classification task by integrating local detail enhancement, morphological relation modeling, and stable discriminative constraints. First, a convolutional residual local detail enhancement front-end is introduced to strengthen fine-grained information such as nuclear boundaries, staining textures, and local heterogeneity, while a Transformer backbone is employed to model cross-regional contextual semantics, thereby establishing a more effective collaboration between local representation and global understanding. Then, to address the insufficient characterization of structural dependencies among cellular regions in traditional feature extraction methods, a morphological relation modeling mechanism is further introduced to explicitly enhance correlation representation among different regions and organizational pattern perception capability. On this basis, from the perspective of stable discriminative learning, a causal-invariant discrimination module is designed to reduce the interference of background variation, staining bias, and non-structural noise on classification decisions, enabling the model to focus more intensively on discriminative information intrinsically related to cell categories. These designs allow the model not only to learn multi-scale phenotypic features of pathological cells at the visual level, but also to improve the reliability and consistency of cytological recognition from the perspectives of structural relationships and stable semantics. Compared with existing studies that mainly emphasize local texture extraction or global semantic modeling, the proposed framework further clarifies the functional division of the three key modules: local detail enhancement addresses the insufficient representation of fine-grained pathological cues, morphological relation modeling addresses the inadequate characterization of inter-regional structural dependencies, and causal-invariant discrimination addresses the instability caused by staining variation, background noise, and imaging bias. Through their progressive collaboration, the model forms a more complete discriminative pathway from local detail perception to structural relationship enhancement and then to robust classification.

The main contributions of this study are as follows:

- To address the problem of insufficient fine-grained local discriminative cues in the glioma cell versus normal cell classification task, a feature encoding framework combining a convolutional residual local detail enhancement front-end with a Transformer backbone is constructed, thereby improving the model’s representation capability for key pathological details such as nuclear boundaries, staining textures, and local heterogeneity. This design mainly improves the sensitivity of the model to subtle cytological differences at the local visual level.
- To address the limitation of traditional methods in modeling morphological organizational relationships among cellular regions, a morphological relation modeling mechanism is designed. By explicitly characterizing the correlations and structural dependencies among different regions, the proposed mechanism enhances the model’s perception capability for complex cytological morphological differences and organizational pattern variations. This mechanism further shifts the model from independent texture response learning toward structure-aware morphological relationship representation.
- To cope with non-essential disturbances commonly existing in pathological images, such as staining variation, background noise, and imaging bias, a causal-invariant discrimination module is proposed to strengthen stable discriminative feature extraction and suppress the influence of incidental statistical correlations on classification decisions, thereby providing a new perspective for intelligent glioma cell recognition that jointly considers fine-grained cytological representation, structural relation modeling, and robust discriminative capability. Together with the local detail enhancement and morphological relation modeling modules, this module enables the overall framework to improve classification performance through complementary enhancement of local, structural, and stable semantic information.

## 2 Materials and methodology

### 2.1 Dataset

This study conducted glioma cell and normal cell classification experiments using retrospective data. The data were collected from pathological image records accumulated during previous clinical diagnosis and treatment processes at our hospital. A total of 473 subjects were included, among whom 201 were patients with glioma and the remaining 272 were non glioma cases. After data organization, a total of 859 pathological images were obtained, and all images were used for subsequent data cleaning, annotation review, and model construction. To ensure annotation quality and consistency of class determination, all images were independently reviewed, scored, and annotated by two experienced physicians. In cases of disagreement, further re evaluation was performed to obtain the final labels. The overall composition of the dataset is shown in Table 1.

**TABLE 1:** Dataset statistics.

Item	Count
Total number of subjects	473
Number of glioma cases	201
Number of non glioma cases	272
Total number of pathological images	859
Number of annotating physicians	2
Study type	Retrospective study
Ethics approval number	2025 review 28

To ensure the reliability and reproducibility of the experimental evaluation, the dataset was divided at the patient level into training, validation, and test sets according to a ratio of 70%, 15%, and 15%, respectively. A stratified random partitioning strategy was adopted to maintain a relatively consistent distribution of glioma and non glioma cases across different subsets. All pathological images from the same subject were assigned to only one subset to avoid patient-level information leakage between training and testing. In the repeated experiments, the patient-level stratified partitioning process was re-executed using different random seeds, and the subject assignments were changed accordingly while maintaining the same partition ratio. Therefore, the repeated experimental results reflect the stability of the model under different random data partitions. Specifically, the training set contained 331 subjects and 601 pathological images, the validation set contained 71 subjects and 129 pathological images, and the test set contained 71 subjects and 129 pathological images. The detailed partitioning information is shown in Table 2.

**TABLE 2:** Patient-level dataset partitioning used in the experiments.

Subset	Subjects	Glioma cases	Non glioma cases	Images
Training set	331	141	190	601
Validation set	71	30	41	129
Test set	71	30	41	129
Total	473	201	272	859

This study strictly followed the ethical standards for medical research. The research protocol was reviewed and approved by the Medical Ethics Committee of Dazhou Integrated Traditional Chinese and Western Medicine Hospital, with the ethics approval number 2025 Review 28. As this study was retrospective in nature, all data were derived from previously archived records, no additional intervention was involved during the research process, and all subject related information was anonymized. Therefore, the requirement for written informed consent was waived. The entire study was conducted in accordance with the Declaration of Helsinki and relevant medical ethical requirements, and the processes of data organization, annotation, and model analysis were completed under the premise of ensuring subject privacy and compliant data use.

### 2.2 Overall model architecture

**FIGURE 1:**
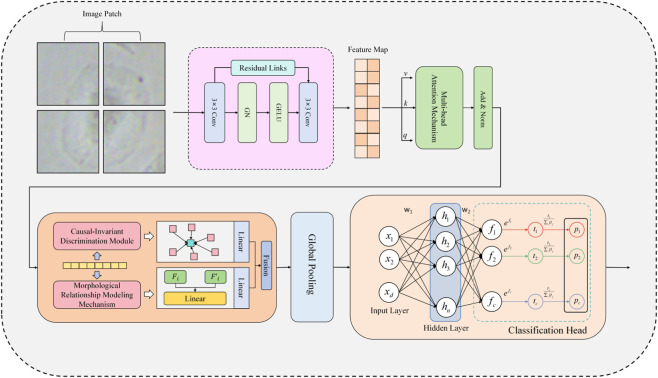
Overall framework of the glioma cell and normal cell classification model. The model first extracts multi scale representations through a convolutional residual local detail enhancement front end and a Swin Transformer backbone. It then performs feature fusion by combining a morphological relationship modeling mechanism with a causal invariant discrimination module, and finally achieves classification through global pooling and a classification head.

This paper focuses on the classification task of glioma cells and normal cells, and constructs a hybrid classification framework based on Swin Transformer that integrates local detail enhancement, morphological relationship modeling, and causal invariant discrimination. The overall pipeline is illustrated in Figure 1.

The input cell image is first divided into several image patches and then fed into a convolutional residual local detail enhancement front end, so that fine grained visual cues such as nuclear boundaries, staining textures, local heterogeneity, and microscopic structural variations can be sufficiently captured at the early stage of feature encoding. This front end consists of two 3×3 convolutional layers, a normalization unit, and a nonlinear activation unit, while a residual connection is introduced to preserve the original local responses, thereby enhancing the feature representation without destroying the underlying structural information. Let the input image patch be denoted as $X\in R^{H\times W\times C}$ . The local representation after convolutional residual enhancement as shown in Formula 1.

$$X_{\text{loc}}=X+{Conv}_{3\times 3}\left(GELU\left(GN\left({Conv}_{3\times 3}\left(X\right)\right)\right)\right)$$ (1)

Subsequently, the enhanced local representation is mapped into sequential features and fed into the Transformer backbone. Through the multi head self attention mechanism, cross region contextual dependencies are modeled, thereby producing feature representations that jointly encode local textures and global semantics. Let the linearly projected query, key, and value be denoted as Q , K , and V , respectively. The multi head attention process in the backbone can be expressed as shown in Formula 2

$$MHSA\left(Q,K,V\right)=Concat\left(H_{1},H_{2},\ldots ,H_{M}\right)W^{O},\;H_{m}=Softmax\left(\frac{Q_{m}K_{m}^{⊤}}{\sqrt{d}}\right)V_{m}.$$ (2)

After the Transformer backbone output is obtained, the model further introduces two core innovative branches to improve the discriminative effectiveness for medical cell image classification. The first branch is the morphological relationship modeling mechanism. This branch is not limited to the independent appearance description of a single image patch, but instead performs relational enhancement and interactive modeling from the perspective of structural connections among cell regions, coordinated variations among local regions, and correlated responses among different morphological components. Considering that glioma cells and normal cells usually exhibit differences in nuclear morphology, cytoplasmic distribution, texture continuity, and local spatial organization, this mechanism can explicitly strengthen morphologically dependent information with pathological significance, allowing the model to evolve from merely focusing on texture intensity to simultaneously capturing morphological organization patterns. Let the backbone output feature be denoted as $F\in R^{N\times D}$ . The representation after morphological relationship modeling is written as shown in Formula 3

$$F_{\text{morph}}=\phi \left(AFW_{r}\right)$$ (3)

where $A\in R^{N\times N}$ denotes the relationship matrix constructed from regional similarity or structural associations, $W_{r}$ denotes the linear transformation parameter, and ϕ(⋅) denotes the nonlinear mapping. Through this process, the structural correlations implicitly contained in the original features are further made explicit, thereby enhancing the model’s ability to perceive cellular morphological differences.

The second branch is the causal invariant discrimination module. This branch is designed to extract discriminative information that is intrinsically related to the class and remains stable across different samples and imaging conditions under complex appearance variations. Compared with feature learning methods that rely solely on statistical correlations, this module places more emphasis on the intrinsic discriminative factors that can stably support the distinction between glioma cells and normal cells, thereby suppressing the interference caused by staining fluctuations, background noise, imaging bias, and non pathological disturbances. Let the output of this branch be denoted as $F_{\text{causal}}$ . The model then performs linear mapping and fusion between this output and the output of the morphological relationship branch, followed by global pooling and a classification head to produce the final prediction. The fused representation can be written as shown in Formula 4

$$F_{\text{fus}}=\psi \left(W_{m}F_{\text{morph}}+W_{c}F_{\text{causal}}\right)$$ (4)

where $W_{m}$ and $W_{c}$ denote the linear mapping parameters corresponding to the two branches, respectively, and ψ(⋅) denotes the fusion function. Based on the above design, the model forms a unified framework composed of a convolutional residual local enhancement front end, a Transformer backbone, a morphological relationship modeling mechanism, and a causal invariant discrimination module. As a result, the classification process simultaneously possesses local detail sensitivity, global contextual modeling capability, structural relationship representation ability, and stable discriminative capability, making it more suitable for the fine grained recognition task of glioma cells and normal cells.

### 2.3 Morphological relationship modeling mechanism

**FIGURE 2:**
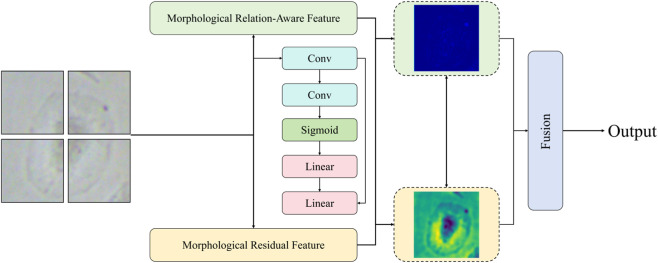
The detailed process of the morphological relationship modeling mechanism. This module extracts morphology relation aware features and morphology residual features from the backbone features in parallel, and strengthens the expression of structural correlations among cell regions as well as the ability to preserve local details through a fusion operation.

After the convolutional residual local detail enhancement front end and the Swin Transformer backbone encoding are completed, let the backbone output feature representation be denoted as $F\in R^{N\times D}$ , where N represents the number of feature tokens and D represents the channel dimension. Although this representation already contains both local texture information and global contextual information, for the classification task of glioma cells and normal cells, relying only on the statistical features produced by conventional Transformer encoding is still insufficient to fully characterize the morphological correlations among cell regions, boundary continuity, and the coordinated variations of local tissue structures. To address this issue, a morphological relationship modeling mechanism is further constructed in the overall framework to perform structure aware enhancement on the backbone output features. Its module architecture is shown in Figure 2.

Intuitively, this module is designed to compensate for the limitation of conventional feature extraction methods that mainly emphasize local texture responses while insufficiently considering the structural relationships among different cellular regions. In pathological cell images, discriminative information is not only reflected in isolated local appearances, such as staining intensity or boundary texture, but also in the coordinated morphological patterns formed by nuclear shape, cytoplasmic distribution, contour continuity, and neighboring regional organization. Therefore, by constructing an explicit relationship matrix among feature tokens, the proposed module enables the model to capture inter-regional dependencies and morphology-aware structural correlations, thereby providing more informative evidence for distinguishing glioma cells from normal cells.

Starting from the same input feature set F , this module simultaneously generates morphology relation aware features and morphology residual features, and then forms a more discriminative structural representation through subsequent fusion. First, the input features are linearly mapped to obtain the basic morphological embedding as shown in Formula 5:

$$Z=FW_{z}+b_{z}$$ (5)

where $W_{z}\in R^{D\times D_{m}}$ and $b_{z}\in R^{D_{m}}$ denote the mapping weight and bias term, respectively, and $D_{m}$ denotes the dimension of the morphological representation space. Based on this embedding, the morphological correlations among different tokens are further computed so as to explicitly characterize the structural dependency relationships among local regions as shown in Formula 6:

$$A=Softmax\left(\frac{ZZ^{⊤}}{\sqrt{D_{m}}}\right)$$ (6)

where $A\in R^{N\times N}$ denotes the morphological relationship matrix, and each element in the matrix reflects the correlation strength among different regions in the morphological space. Through this process, the implicit structural relationships contained in the original features are transformed into a relational representation that can be explicitly modeled, thereby providing the basis for subsequent morphological enhancement.

After the morphological relationship matrix is obtained, it is applied to the input features to extract morphology relation aware features. This branch mainly emphasizes the structural coordination information among different regions, so that the model is no longer limited to the independent responses of individual local textures, but can simultaneously focus on cell contour continuity, perinuclear texture organization, and the interdependence among local regions. The morphology relation aware feature is defined as shown in Formula 7

$$F_{\text{rel}}=\sigma \left(AFW_{r}+b_{r}\right)$$ (7)

where $W_{r}\in R^{D\times D}$ and $b_{r}\in R^{D}$ denote the transformation parameters of the relation branch, respectively, and σ(⋅) denotes the nonlinear activation function. Meanwhile, in order to avoid weakening the original local discriminative details in the input features during relation propagation, the module further preserves a morphology residual branch, which forms residual features by performing linear projection on the input features as shown in Formula 8:

$$F_{\text{res}}=FW_{s}+b_{s}$$ (8)

where $W_{s}\in R^{D\times D}$ and $b_{s}\in R^{D}$ denote the parameters of the residual branch, respectively. This design enables the relation modeling branch to highlight the morphological relationships among regions, while the residual branch preserves the original responses such as local cellular structures, texture intensity, and edge details, thereby allowing the model to achieve a balance between structural relationship modeling and local information preservation.

On this basis, the morphology relation aware features and morphology residual features are fused to form the final output of morphological relationship modeling, which is then used as an important input to the subsequent causal invariant discrimination module and the overall fusion module. The fused morphological representation is denoted as shown in Formula 9

$$F_{\text{morph}}=\phi \left(W_{f}^{\left(1\right)}F_{\text{rel}}+W_{f}^{\left(2\right)}F_{\text{res}}+b_{f}\right)$$ (9)

where $W_{f}^{\left(1\right)}$ and $W_{f}^{\left(2\right)}$ denote the fusion weights corresponding to the two branches, $b_{f}$ denotes the bias term, and ϕ(⋅) denotes the nonlinear mapping function after fusion. The resulting $F_{\text{morph}}$ is consistent with the morphological feature branch defined in the overall model architecture in the previous subsection, and will subsequently participate in feature integration together with the output $F_{\text{causal}}$ of the causal invariant discrimination branch. Compared with directly using the backbone output feature F for classification, this mechanism can model more explicitly the differences between glioma cells and normal cells in terms of morphological organization patterns, so that the network in the classification decision process not only focuses on texture intensity and local appearance, but also further exploits the structural relationship information among regions to improve the recognition ability for complex cellular morphologies. Therefore, the design value of this module lies in guiding the model to move beyond independent local texture recognition and toward structure-aware cytological pattern understanding, which is particularly important for pathological image classification where morphological organization often carries strong diagnostic significance.

### 2.4 Causal-invariant discrimination module

**FIGURE 3:**
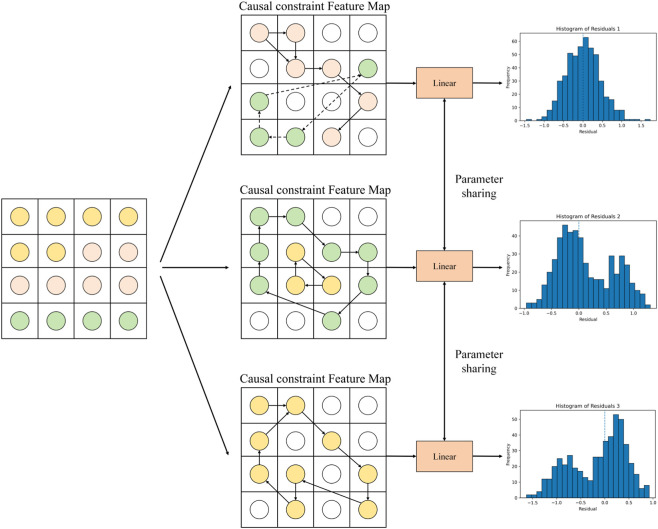
The detailed process of the causal invariant discrimination module. This module extracts stable discriminative representations through multi path causal constraint feature mapping and a parameter sharing strategy, and combines residual distribution analysis to suppress non essential disturbance information, thereby enhancing the robustness and consistency of the model for the classification of glioma cells and normal cells.

After the convolutional residual local detail enhancement front end and the Swin Transformer backbone encoding are completed, the backbone output feature is denoted as $F\in R^{N\times D}$ , where N denotes the number of tokens and D denotes the channel dimension. Furthermore, in the previous subsection, the morphological relationship modeling mechanism has already been constructed based on F , yielding the morphology enhanced representation $F_{\text{morph}}\in R^{N\times D}$ . However, for the classification task of glioma cells and normal cells, relying only on morphological structural differences may still be affected by non essential factors such as staining fluctuations, background texture disturbances, local noise, and changes in imaging conditions, which may further cause the model to mistake accidental co occurring statistical patterns for stable discriminative evidence. To address this issue, this paper further introduces a causal invariant discrimination module into the overall framework. While maintaining collaborative interaction with the morphological relationship modeling branch, this module extracts more stable and class essential discriminative representations from the backbone features, and its architecture is illustrated in Figure 3.

Considering that the morphological branch has already enhanced structural dependencies, this module takes the backbone output F as the basis and introduces the morphological prior $F_{\text{morph}}$ as an auxiliary constraint, so as to first construct a joint input representation as shown in Formula 10:

$$F_{\text{joint}}=\alpha F+\left(1-\alpha \right)F_{\text{morph}}$$ (10)

where α∈[0,1] is the balancing coefficient. In order to further obtain a basic embedding for causal constraint modeling, the joint representation is mapped into a latent discriminative space, which is defined as shown in Formula 11

$$Z_{\text{c}}=F_{\text{joint}}W_{c}^{\left(0\right)}+b_{c}^{\left(0\right)}$$ (11)

where $W_{c}^{\left(0\right)}\in R^{D\times D_{c}}$ and $b_{c}^{\left(0\right)}\in R^{D_{c}}$ denote the linear transformation parameters, respectively, and $D_{c}$ denotes the dimension of the causal representation space. This representation provides a unified input for the subsequent multi path causal constraint mapping and invariant discrimination modeling.

To reduce the representation bias caused by single path feature selection, the causal invariant discrimination module adopts a multi path parallel mapping strategy, which projects $Z_{\text{c}}$ into several subspaces with shared parameter constraints, thereby extracting stable discriminative patterns from different perspectives. Suppose that the module constructs a total of K parallel causal constraint paths. The feature representation corresponding to the k th path is as shown in Formula 12

$$Z_{k}=Z_{\text{c}}W_{k}+b_{k},\;k=1,2,\ldots ,K$$ (12)

where $W_{k}\in R^{D_{c}\times D_{c}}$ and $b_{k}\in R^{D_{c}}$ denote the mapping parameters of the k th path. In order to keep the paths aligned toward consistent discriminative directions under different local disturbances, a parameter sharing constraint is further introduced across paths. Specifically, the core linear transformation of each path is modeled around a shared base matrix, which can be written as shown in Formula 13

$$W_{k}=W_{\text{share}}+\Delta W_{k}$$ (13)

where $W_{\text{share}}$ denotes the shared discriminative base matrix, and $\Delta W_{k}$ denotes the adaptive offset of the k th path relative to the shared parameters. Based on this design, the module not only preserves the diverse modeling capability of different paths for potential discriminative patterns, but also suppresses unstable responses caused by excessive divergence among paths through the parameter sharing mechanism, allowing different constraint paths to focus more consistently on the common discriminative factors related to class essence. For the cell classification task, this means that although different paths may attend to different aspects such as textures, morphological boundaries, or local regional differences, they are ultimately constrained toward the same stable discriminative semantics.

After obtaining the multi path causal constraint features, the module further separates the stable components and disturbance components in each path so as to reduce the interference of class irrelevant appearance bias on classification decisions. Considering that the input features may simultaneously contain class essential information and environment related information, the response of the k th path is decomposed into a stable discriminative part and a residual disturbance part, namely as shown in Formulas 14, 15,

$$S_{k}=\sigma \left(Z_{k}W_{k}^{s}+b_{k}^{s}\right)$$ (14)

$$R_{k}=Z_{k}-S_{k}$$ (15)

where $S_{k}$ denotes the stable discriminative representation extracted from the k th path, $R_{k}$ denotes the residual disturbance component that is not absorbed by the stable discriminative mapping, $W_{k}^{s}$ and $b_{k}^{s}$ are the corresponding mapping parameters, and σ(⋅) denotes the nonlinear activation function. Since the residual term mainly reflects unstable factors such as local noise, background changes, staining differences, and nonstructural disturbances, the module further constructs a path confidence weight based on the residual magnitude to dynamically adjust the contribution ratio of different paths in the final discrimination. Let the residual statistic of the k th path be as shown in Formula 16

$$r_{k}=\frac{1}{ND_{c}}{\left\|R_{k}\right\|}_{1}$$ (16)

then the corresponding path weight is defined as shown in Formula 17

$$\beta_{k}=\frac{\exp\left(-r_{k}\right)}{\sum_{j=1}^{K}\exp\left(-r_{j}\right)}$$ (17)

It can thus be seen that paths with smaller residuals are more likely to preserve stable and consistent discriminative factors, and will therefore receive larger weights in the final fusion, whereas paths with larger residuals contain more disturbance components and their contributions will be automatically weakened. This mechanism is complementary to the structure explicit modeling emphasized by the morphological relationship modeling module in the previous subsection, that is, the morphological branch is responsible for highlighting cellular organizational relationships and regional coordination features, while the causal branch further filters out unstable responses caused by non essential fluctuations on this basis.

Finally, the stable discriminative representations of all paths are aggregated with weights to form the final output of the causal invariant discrimination module, while maintaining a unified variable interface with the morphological feature representation $F_{\text{morph}}$ defined in the previous subsection so that it can be incorporated into the fusion module in the overall framework. Specifically, the causal invariant representation is defined as shown in Formula 18

$$F_{\text{causal}}=\overset{K}{\underset{k=1}{\sum }}\beta_{k}S_{k}$$ (18)

To further improve feature scale consistency and representation stability, a normalization mapping is applied to the aggregated result, yielding as shown in Formula 19

$${\tilde{F}}_{\text{causal}}=Norm\left(F_{\text{causal}}W_{o}+b_{o}\right)$$ (19)

where $W_{o}$ and $b_{o}$ are the output mapping parameters, and Norm(⋅) denotes the normalization operation. The resulting ${\tilde{F}}_{\text{causal}}$ is exactly the output of the causal invariant discrimination branch used in the overall model architecture subsection. In the subsequent stage, it will be jointly fed into the fusion module together with $F_{\text{morph}}$ to form a highly discriminative joint representation for global pooling and classification head prediction. Compared with directly relying on the backbone feature F or performing classification only based on the morphology enhanced result $F_{\text{morph}}$ , this module further strengthens the model’s ability to focus on the class essential characteristics of glioma cells through the multi path shared mapping, residual separation, and stability weighting mechanism, enabling the network to maintain stronger discriminative consistency and generalization stability even in the presence of complex backgrounds, staining differences, and local noise interference.

### 2.5 Experimental setup

Model training and testing in this study were conducted on an NVIDIA A100 80 GB GPU platform. The experimental environment was built on the Linux operating system, and the deep learning framework was implemented using PyTorch. To ensure training stability and result reproducibility for the classification task of glioma cells and normal cells, a unified setting of input size, batch size, optimizer, and learning rate scheduling strategy was adopted throughout the experiments, and the model parameters were optimized in an end to end manner during training. Specifically, the input images were first resized to a unified scale and then fed into the network. The AdamW optimizer was used, with the initial learning rate set to $1\times 10^{-4}$ , the weight decay coefficient set to $1\times 10^{-5}$ , the batch size set to 16, and the number of training epochs set to 100. Meanwhile, a cosine annealing strategy was employed to dynamically adjust the learning rate so as to improve the stability of model convergence in the later stage. In the repeated experiments, the random seeds controlled not only model initialization and training randomness, but also the patient-level stratified data partitioning process. Therefore, the reported mean and standard deviation reflect the stability of the model under different random data partitions and training initializations. The main software and hardware environment and training hyperparameter settings involved in the experiments are shown in Table 3.

**TABLE 3:** Experimental software and hardware environment and main hyperparameter settings.

Item	Setting
Operating system	Linux
GPU	NVIDIA A100 80 GB
Programming language	Python 3.10
Deep learning framework	PyTorch 2.1
CUDA version	CUDA 12.1
Input image size	224×224
Batch size	16
Training epochs	100
Optimizer	AdamW
Initial learning rate	$1\times 10^{-4}$
Weight decay	$1\times 10^{-5}$
Learning rate scheduling strategy	Cosine annealing

## 3 Experimental results and analysis

### 3.1 Comparison of experimental results with other models

To further validate the effectiveness and overall performance of the proposed method in the classification task of glioma cells and normal cells, this study selected multiple representative deep learning models as comparative methods and conducted a systematic comparison from multiple perspectives, including classification accuracy, discriminative ability, and inference efficiency. The detailed comparative experimental results are presented in Table 4, where Acc, Precision, Recall, and AUC are reported as the mean and standard deviation over three repeated experiments with different random seeds, while FPS is used to characterize the inference speed of each model.

**TABLE 4:** Comparison of experimental results with other models. The values of Acc, Precision, Recall, and AUC are reported as mean ± standard deviation over three runs with different random seeds, where each seed controls both patient-level data partitioning and model initialization.

Method	Acc	Precision	Recall	AUC	FPS
ResNet50 He et al. (2016)	94.81 ± 0.34	93.96 ± 0.41	94.22 ± 0.39	0.952 ± 0.004	146.8
VGG19 Simonyan and Zisserman (2014)	93.92 ± 0.39	93.21 ± 0.45	93.47 ± 0.42	0.944 ± 0.005	98.4
ConvNextv2 Woo et al. (2023)	95.37 ± 0.31	94.88 ± 0.36	94.96 ± 0.33	0.961 ± 0.003	118.7
ResNext Xie et al. (2017)	95.14 ± 0.28	94.42 ± 0.34	94.71 ± 0.31	0.958 ± 0.004	132.6
Vision-transformer Dosovitskiy et al. (2020)	95.83 ± 0.30	95.12 ± 0.32	95.26 ± 0.29	0.966 ± 0.003	87.2
EFFResNet-ViT Hussain et al. (2025)	96.08 ± 0.27	95.46 ± 0.30	95.61 ± 0.28	0.968 ± 0.003	92.5
Conv-SdMLPMixer Ren et al. (2025)	95.42 ± 0.35	94.97 ± 0.37	95.03 ± 0.34	0.962 ± 0.004	110.3
Medkan Yang et al. (2025b)	94.65 ± 0.40	94.06 ± 0.43	94.11 ± 0.39	0.951 ± 0.005	121.4
Nqnn Rahman and Zhuang (2025)	93.58 ± 0.43	92.91 ± 0.46	93.05 ± 0.44	0.941 ± 0.006	154.9
TopoImages Gu et al. (2025)	95.96 ± 0.29	95.29 ± 0.31	95.41 ± 0.30	0.967 ± 0.003	104.8
PHOG-Net Al-Wajih et al. (2026)	94.22 ± 0.37	93.56 ± 0.40	93.74 ± 0.38	0.947 ± 0.005	139.7
Proto-Caps Gallée et al. (2025)	95.01 ± 0.33	94.36 ± 0.35	94.58 ± 0.32	0.956 ± 0.004	89.6
CARE Yang et al. (2025a)	96.21 ± 0.25	95.63 ± 0.27	95.74 ± 0.26	0.969 ± 0.003	101.9
kMaXU Huang et al. (2025)	95.68 ± 0.31	95.05 ± 0.33	95.13 ± 0.30	0.964 ± 0.004	96.3
AMIAC Iqbal et al. (2025)	96.43 ± 0.24	95.88 ± 0.26	96.02 ± 0.25	0.971 ± 0.003	108.1
**Ours**	**98.46** ± **0.18**	**97.94** ± **0.20**	**98.07** ± **0.19**	**0.987** ± **0.002**	**116.5**

Bold text indicates optimality.

From the overall results, the proposed method achieved the best performance on all four core metrics, namely Acc, Precision, Recall, and AUC, reaching 98.46%, 97.94%, 98.07%, and 0.987, respectively. Compared with different types of baseline models, including convolutional networks, Transformer-based models, and hybrid medical image classification architectures, the proposed method showed consistent performance advantages. These results indicate that the constructed classification framework not only improves the overall discrimination accuracy between glioma cells and normal cells, but also demonstrates stronger advantages in positive sample recognition, class separation stability, and comprehensive discriminative capability. Further analysis of the standard deviation values shows that the proposed method still maintained small fluctuations across three repeated experiments with different random seeds, where the standard deviations of Acc, Precision, Recall, and AUC were only 0.18, 0.20, 0.19, and 0.002, respectively. This finding suggests that the model not only has a high performance upper bound, but also exhibits favorable training stability and result consistency. Such advantages indicate that the proposed method does not rely merely on a single local texture or incidental statistical correlation for classification, but is capable of learning more stable and more intrinsically meaningful discriminative features under complex cellular morphological variations and microscopic image disturbances.

To make the comparative results more intuitive, the methods in Table 4 can be understood from three representative dimensions. ResNet50, VGG19, ConvNextv2, and ResNeXt mainly represent convolutional network-based methods, Vision Transformer represents global attention-based modeling, while EFFResNet-ViT, CARE, AMIAC, and related models represent hybrid or medical image-oriented architectures. Further insights can be obtained by examining the model design, where the performance improvement is closely associated with several key innovative modules introduced in this study. On the one hand, the combination of the convolutional residual local detail enhancement front end and the Swin Transformer backbone enables the model to simultaneously capture fine grained information, such as nuclear boundaries, staining textures, and local heterogeneity, as well as cross regional contextual semantics, thereby establishing a more sufficient feature foundation for subsequent discrimination. On the other hand, the morphological relation modeling mechanism further strengthens the representation of structural associations among cellular regions, local tissue coordination, and morphological organization patterns, allowing the network to move beyond independent texture responses and capture region relationship features with greater diagnostic value from the perspective of pathological morphology. On this basis, the causal invariant discrimination module further suppresses the influence of non essential interference factors, including staining variations, background noise, and imaging bias, on classification decisions through multi path shared mapping, residual separation, and stability weighting mechanisms. Therefore, while maintaining relatively high inference efficiency, the proposed method can more effectively highlight stable discriminative information that is intrinsically related to class essence, which is also the key reason why it achieves better results than various convolutional models, Transformer based models, and hybrid architecture models.

### 3.2 Ablation test results

To further verify the practical contribution of each key component in the proposed model to the classification task of glioma cells and normal cells, this study conducted a stepwise ablation analysis around the core modules in the overall framework and established a comparative experimental setting from the baseline model to the complete model. The detailed ablation results are presented in Table 5, where all metrics are reported as the mean and standard deviation over three repeated experiments with different random seeds.

**TABLE 5:** Ablation experiment results of different model components on the classification task of glioma cells and normal cells.

Ablation setting	Acc	Precision	Recall	AUC
Baseline	95.92 ± 0.27	95.31 ± 0.30	95.44 ± 0.28	0.966 ± 0.003
+ Convolutional residual local detail enhancement front end	96.63 ± 0.22	96.04 ± 0.34	96.17 ± 0.26	0.972 ± 0.005
+ Morphological Relationship Modeling Mechanism	96.38 ± 0.31	95.86 ± 0.19	95.94 ± 0.27	0.970 ± 0.002
+ Causal Invariant Discrimination Module	97.96 ± 0.18	97.39 ± 0.28	97.52 ± 0.17	0.983 ± 0.004
Full model	98.46 ± 0.18	97.94 ± 0.20	98.07 ± 0.19	0.987 ± 0.002

The ablation results show that, starting from the baseline model, different modules contribute differently to the classification performance of glioma cells and normal cells. Among them, the introduction of the convolutional residual local detail enhancement front end can steadily improve Acc, Precision, Recall, and AUC, indicating that this module can effectively strengthen the representation of fine grained visual cues such as nuclear boundaries, staining textures, and local heterogeneity, thereby providing more sufficient low level feature support for subsequent classification. In comparison, although the introduction of the morphological relationship modeling mechanism alone also brings a certain degree of performance improvement, its overall gain is slightly lower than that of the local detail enhancement front end, suggesting that when only structural associations among regions are modeled, the supplementation of local discriminative details remains limited. After the causal invariant discrimination module is incorporated, the model performance is further improved significantly, indicating that this module can more effectively suppress non essential interference information such as staining variations, background noise, and imaging bias, while highlighting stable features that are directly related to class discrimination. Ultimately, the full model achieves the best results on all evaluation metrics, demonstrating that the proposed convolutional residual local detail enhancement, morphological relationship modeling, and causal invariant discrimination are not isolated from each other, but instead collaboratively enhance the recognition capability and overall robustness of the model for complex pathological cell images through a progressive synergistic mechanism that moves from local detail representation to structural relationship reinforcement and then to stable discriminative constraint.

### 3.3 Visualize experimental results

#### 3.3.1 t-SNE experimental results

**FIGURE 4:**
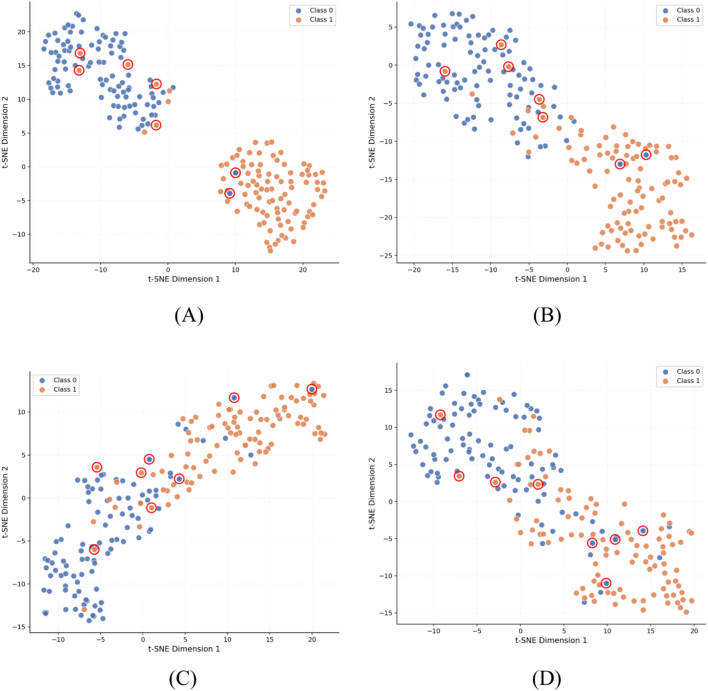
The t-SNE visualization results of the proposed method and the three best performing comparative models show that, compared with the comparative methods, the proposed method exhibits clearer class separation boundaries, higher intra class compactness, and fewer misclassified sample distributions in the classification task of glioma cells and normal cells. **(A)** Ours, **(B)** EFFResNet-ViT, **(C)** Topolmages, **(D)** AMIAC.

To further provide an intuitive analysis of the differences in feature distribution among different models in the classification task of glioma cells and normal cells, this study employed the t-SNE method to perform dimensionality reduction visualization of high dimensional features. In addition to the proposed method, the three comparative models with the best overall performance were further selected for visual comparison, so as to more clearly demonstrate the differences among different methods in terms of class aggregation capability, degree of feature separation, and the distribution of misclassified samples. The experimental results are shown in Figure 4.

The t-SNE visualization results show that the proposed method forms a clearer and more compact intra class aggregation structure in the classification task of glioma cells and normal cells, while maintaining more distinct separation boundaries between different classes, which indicates that the learned features possess stronger discriminative ability and greater stability. In comparison, although EFFResNet-ViT and AMIAC can achieve class discrimination to a certain extent, there are still problems of sample overlap in some boundary regions and relatively scattered distributions of misclassified points, whereas the feature mixing phenomenon between the two classes in TopoImages is more obvious, indicating that its ability to represent complex cellular morphological differences is relatively limited. This result is consistent with the structural design of the proposed model, where the convolutional residual local detail enhancement front end helps strengthen the expression of fine grained information such as nuclear boundaries, staining textures, and local heterogeneity, the morphological relationship modeling mechanism further enhances the representation capability of structural associations among regions and organizational patterns, and the causal invariant discrimination module effectively suppresses non essential interference factors such as staining variations, background noise, and imaging bias. As a result, the model is able to learn more stable feature representations with stronger class essential meaning, ultimately showing better inter class separability and fewer abnormal misclassified sample distributions at the visualization level.

#### 3.3.2 AUROC experimental results

**FIGURE 5:**
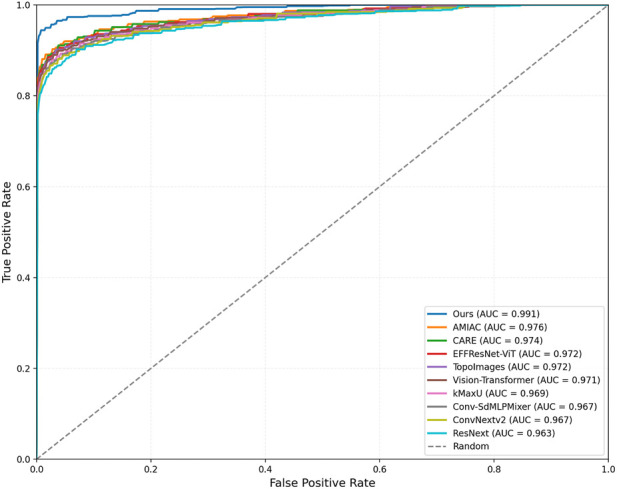
The AUROC curve comparison results of the proposed method and the other nine best-performing comparative models in a representative test run show that the proposed method achieves a curve closer to the upper-left corner and a larger enclosed area, indicating stronger global discriminative ability and better classification stability in glioma cell and normal cell classification.

To further evaluate the overall discriminative ability of different models in the classification task of glioma cells and normal cells, this study adopted AUROC as the core evaluation metric and conducted a unified comparative analysis on the nine models with the best overall performance. The detailed AUROC comparison results are shown in Figure 5. This experiment can more intuitively reflect the differences among models in class discrimination ability and classification stability across the overall threshold variation range.

The experimental results show that the ROC curve of the proposed method is overall closer to the upper left corner, and the corresponding AUC value is the highest, indicating that it can maintain superior positive recognition ability and class discrimination capability under different classification thresholds, thereby demonstrating stronger overall robustness and generalization stability. In comparison, although the other comparative models can also achieve favorable classification performance, their curves still show different degrees of downward shift in the low false positive rate region and in the medium to high threshold range, suggesting that their discrimination of complex samples and boundary samples remains more susceptible to feature entanglement and non essential disturbances. This result further verifies the effectiveness of the proposed method. Specifically, the convolutional residual local detail enhancement front end can fully extract key fine grained information such as nuclear boundaries, staining textures, and local heterogeneity, the morphological relationship modeling mechanism further strengthens the structural associations among different regions and the representation of pathological organizational patterns, and the causal invariant discrimination module enables the model to focus more effectively on stable discriminative features that are intrinsically related to the class essence of glioma cells by suppressing the interference of non critical factors such as background noise, staining variations, and imaging bias. Therefore, the proposed method achieves a more obvious advantage in overall AUROC performance.

### 3.4 Confusion matrix experimental results

**FIGURE 6:**
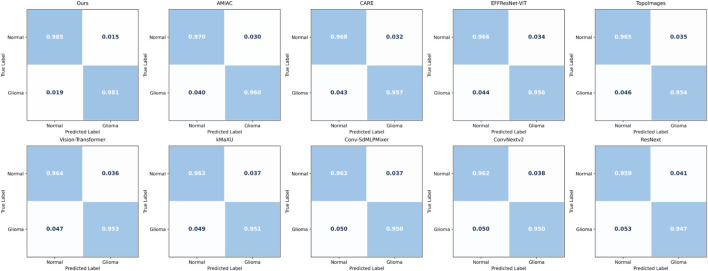
The normalized confusion matrix visualization results of different models on the glioma cell versus normal cell classification task show that the proposed method achieves higher discriminative accuracy and lower misclassification rates for both categories.

To further provide an intuitive analysis of the recognition behaviors of different methods in the glioma cell versus normal cell classification task from the perspective of class-discriminative structure, confusion matrices were introduced to visualize the prediction distributions of each model over the two categories. Under the premise of maintaining consistency with the overall classification task setting, this part presents a unified comparison of the ten best-performing methods, so as to more clearly reveal the differences among models in terms of class separation tendency and misclassification patterns, as shown in Figure 6.

From the overall distribution of the normalized confusion matrices, it can be observed that the proposed method exhibits a significantly higher proportion in the diagonal regions for both glioma cell and normal cell samples, while maintaining lower misclassification proportions in the off-diagonal regions, indicating stronger class discrimination capability and more stable decision consistency during positive and negative class recognition. Compared with the other competing models, the proposed method not only preserves a higher correct classification rate for normal cells, but also demonstrates a lower miss classification rate for glioma cells, which is the clinically critical category. This suggests that the constructed convolutional residual local detail enhancement front-end effectively strengthens fine-grained discriminative information such as nuclear boundaries, staining textures, and local heterogeneity, while the morphological relation modeling mechanism further improves the representation ability of structural correlation features among different regions. In addition, the causal-invariant discrimination module helps suppress non-essential interferences, including background noise, staining variation, and imaging bias, thereby enabling the model to focus more consistently on discriminative evidence that is intrinsically related to category semantics. As a result, the proposed method presents a superior overall classification structure and clearer category separation in the confusion matrix analysis.

### 3.5 Confidence distribution results

**FIGURE 7:**
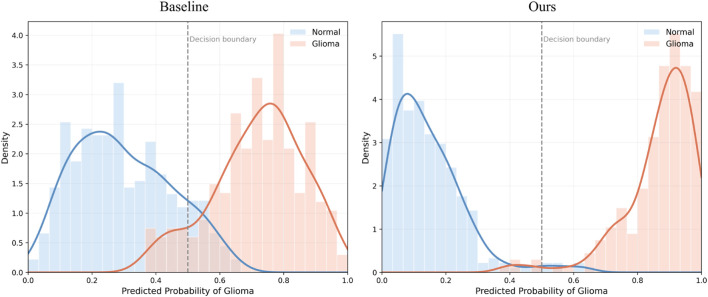
The visualization results of the prediction confidence distributions of the baseline model and the proposed method show that the proposed method yields a more separable probability distribution for the two categories, with fewer ambiguous samples located near the decision boundary.

To further provide an intuitive analysis of the discriminative behaviors of different methods in the glioma cell versus normal cell classification task from the perspective of prediction confidence, the category probability distributions output by the models were visualized. Under a unified classification setting, this part compares the prediction confidence distribution characteristics of the baseline model and the proposed method, so as to more clearly characterize the differences between different methods in terms of class separability and boundary-sample representation. Through this visualization process, the stability of model discrimination for the two categories can be further examined from the perspective of distribution structure, and the corresponding experimental results are shown in Figure 7.

From the prediction confidence distributions, it can be observed that under the baseline model, the probability distributions of normal cells and glioma cells still exhibit a certain degree of overlap, especially around the decision boundary where a relatively large number of uncertain samples remain, indicating that its discriminative stability for boundary samples is still limited. In contrast, under the proposed method, the predicted probabilities of the two categories are more clearly concentrated toward the high-confidence interval of their respective classes, where normal cell samples are distributed more closely to the low-probability region and glioma cell samples are more concentrated near the high-probability region. Meanwhile, the proportion of samples in the intermediate transition region is significantly reduced, suggesting that the model can more effectively enlarge the confidence margin between different categories and alleviate class confusion. This phenomenon indicates that, through the collaborative effect of the proposed convolutional residual local detail enhancement front-end, morphological relation modeling mechanism, and causal-invariant discrimination module, the model not only improves its representation ability for fine-grained pathological features and structural relational information, but also strengthens its suppression of non-essential disturbance factors, thereby demonstrating higher stability, clearer probability separation structure, and stronger robustness during category discrimination.

### 3.6 Hyperparameter sensitivity experimental results

#### 3.6.1 The impact of morphological embedding dimension of the morphological relationship modeling module on experimental results

To further analyze the influence of the morphological embedding dimensionality in the morphological relation modeling module on the discriminative capability and feature representation effectiveness of the model, a systematic comparison was conducted on the performance variations under different dimensional configurations while keeping all other experimental settings unchanged. This part aims to investigate the relationship between the scale of the morphological representation space and classification performance from the perspective of parameter sensitivity, so as to more clearly reveal the effectiveness and stability of this module in the process of structural relation modeling. Through this analysis, the current parameter setting can be further validated in terms of the balance it achieves between model representation capacity and redundancy control.

As shown in Table 6, with the gradual increase of the morphological embedding dimensionality, the overall model performance exhibits a trend of first improving and then declining. Among all configurations, when the dimensionality is set to 128, Acc, Precision, Recall, and AUC all reach their highest values, indicating that this configuration can more adequately characterize the morphological relationships among different cellular regions while avoiding the insufficient representation caused by overly low-dimensional settings. When the dimensionality is further increased to 256 and 512, all evaluation metrics show a slight decline, suggesting that an excessively large morphological representation space may introduce a certain degree of redundant information and weaken the compactness and discriminative effectiveness of structural relation modeling. Therefore, setting the morphological embedding dimensionality to 128 enables the model to achieve a better balance between feature representation capacity and redundancy control, thereby being more beneficial for improving the overall performance of the glioma cell versus normal cell classification task.

**TABLE 6:** Sensitivity analysis of the morphological embedding dimension in the morphological relationship modeling mechanism.

Morphological embedding dimension	Acc	Precision	Recall	AUC
32	97.68	97.14	97.26	0.979
64	98.07	97.52	97.66	0.983
128	98.46	97.94	98.07	0.987
256	98.21	97.63	97.78	0.984
512	97.94	97.38	97.51	0.981

#### 3.6.2 The impact of the number of parallel branches in the causal-invariant discrimination module on experimental results

To further analyze the influence of the number of parallel branches in the causal-invariant discrimination module on the discriminative capability and stable feature modeling effect of the model, a systematic comparison was conducted on the performance variations under different parallel branch configurations while keeping all other experimental settings unchanged. This part aims to investigate the relationship between multi-path structural complexity and classification performance from the perspective of parameter sensitivity, so as to more clearly reveal the effectiveness and robustness of this module in the process of stable discriminative information extraction and disturbance suppression. Through this analysis, the current setting of the number of parallel branches can be further validated in terms of the balance it achieves between representation capacity and structural redundancy control.

As shown in Table 7, with the increase in the number of parallel branches, the overall model performance exhibits a trend of first improving and then declining. Among all configurations, when the number of parallel branches is set to 4, Acc, Precision, Recall, and AUC all reach their highest values, indicating that a moderate multi-path structure can more adequately mine stable discriminative patterns and enhance the suppression ability for complex disturbance information. When the number of branches is further increased to 5 and 6, all evaluation metrics show a slight decline, suggesting that an excessive number of parallel branches may introduce a certain degree of structural redundancy and information dispersion, thereby weakening the effectiveness of stable feature aggregation. Therefore, setting the number of parallel branches to 4 enables the model to achieve a better balance between diversified discriminative information modeling and redundancy control, thereby being more beneficial for improving the overall performance of the glioma cell versus normal cell classification task.

**TABLE 7:** Sensitivity analysis of the number of parallel branches in the causal-invariant discrimination module.

Number of parallel branches	Acc	Precision	Recall	AUC
1	97.82	97.29	97.41	0.981
2	98.13	97.57	97.71	0.984
3	98.31	97.76	97.89	0.985
4	98.46	97.94	98.07	0.987
5	98.24	97.68	97.82	0.984
6	98.06	97.49	97.63	0.982

### 3.7 The effect of different resolutions on experimental results

To further evaluate the stability of the proposed method under different image scales, additional experiments were conducted by resizing the input pathological images to multiple resolutions while keeping the remaining experimental settings unchanged. This analysis aims to examine whether the model can maintain stable discriminative performance when the amount of fine-grained visual information and background detail varies across different input resolutions. The experimental results are shown in Table 8.

**TABLE 8:** Sensitivity analysis of different input image resolutions.

Input resolution	Acc	Precision	Recall	AUC
128×128	97.42	96.86	97.03	0.978
160×160	97.89	97.31	97.46	0.982
192×192	98.18	97.63	97.79	0.985
224×224	98.46	97.94	98.07	0.987
256×256	98.32	97.78	97.91	0.986
320×320	98.11	97.55	97.72	0.984

The experimental results show that as the input resolution increases from 128×128 to 224×224 , the model achieves gradual improvements in Acc, Precision, Recall, and AUC, indicating that a higher resolution can preserve more nuclear boundary information, staining textures, and local morphological details, thereby enhancing the discriminative capability of the model. When the resolution is further increased to 256×256 and 320×320 , all metrics show a slight decline while still remaining at a high level, suggesting that an excessively high resolution may introduce redundant background information or increase feature complexity without bringing further performance gains. Overall, the model maintains stable performance across different input scales, verifying the robustness of the proposed method to changes in input resolution.

## 4 Discussion

### 4.1 Clinical application value

From the perspective of medical application, pathological cell images usually contain fine-grained texture differences, local structural heterogeneity, and complex background interference. Conventional visual analysis and traditional image-based methods may be affected by staining variation, imaging-condition differences, and non-pathological noise, which can reduce the stability of cell discrimination Lin et al. (2025); Asadi-Aghbolaghi et al. (2024). To address these challenges, the proposed method strengthens key pathological cues, including nuclear boundaries, staining textures, and local heterogeneous patterns, through the convolutional residual local detail enhancement front-end. Meanwhile, the global contextual modeling component further improves the ability to capture long-range dependencies among cellular regions.

These designs enable the model to maintain more stable recognition performance in complex microscopic image environments. From a clinical perspective, this stable classification capability may help reduce the burden of manual preliminary screening and provide auxiliary evidence for the rapid identification of suspicious glioma-related cells. Therefore, the proposed method is not intended to replace pathological diagnosis, but may serve as a computer-aided tool to support more efficient and consistent pathological image review in clinical practice Makhlouf et al. (2025); Wang et al. (2024).

### 4.2 Cytological interpretation and morphological relationship modeling

From the perspective of cytological analysis, an important contribution of this work is that it moves beyond single local texture intensity or superficial visual differences and further emphasizes morphological relationship modeling among cellular regions. The differences between glioma cells and normal cells are not only reflected in local color intensity or texture patterns, but also in nuclear morphology, cytoplasmic distribution, boundary continuity, and structural coordination among different regions. Recent studies on histopathological image analysis have also emphasized that modeling spatial dependencies and regional relationships is important for capturing tissue-level and cell-level structural patternsBrussee et al. (2025).

The proposed method characterizes inter-region correlations through morphological relationship modeling and suppresses non-essential interference, such as staining differences, background noise, and imaging bias, through the causal-invariant discrimination module. In this way, the model is encouraged to focus on discriminative evidence that is more closely related to intrinsic cell-category differences. The combination of morphological relationship modeling and causal-invariant discrimination improves the representation of complex cellular structures and enhances the consistency between model decisions and cytological interpretation logic. This provides a useful reference for subsequent studies on fine-grained pathological cell recognition, cell subtype analysis, and intelligent cytology.

### 4.3 Limitations and future work

Although the proposed method achieves favorable experimental results in glioma cell versus normal cell classification, several limitations remain. First, the current study is based on retrospective data with a relatively limited sample scale. The data also have certain single-center characteristics, which may restrict the generalization capability of the model under broader clinical scenarios and different imaging conditions. Previous studies have shown that domain shifts caused by differences in imaging protocols, devices, staining procedures, and data sources may lead to unstable model performance across external cohorts Asadi-Aghbolaghi et al. (2024); Lin et al. (2025).

Second, the current task mainly focuses on binary classification and has not yet been extended to more complex multi-class cell subtyping or robustness validation across different devices and staining conditions. Therefore, the applicability of the model in more complex cytological applications still requires further evaluation. In addition, although the proposed method improves model performance through morphological relationship modeling and causal-invariant discrimination, the correspondence between the internal discriminative mechanism of the model and pathological prior knowledge can be further strengthened. In future work, multi-center data, more pathological cell categories, and richer clinical prior information will be incorporated to further validate the model. Causal-inspired generalization strategies may also provide useful guidance for reducing the influence of spurious correlations in medical image analysis Wei et al. (2024).

## 5 Conclusion

This study proposes a hybrid classification framework for the glioma cell versus normal cell classification task by integrating a convolutional residual local detail enhancement front-end, a morphological relation modeling mechanism, and a causal-invariant discrimination module. On the basis of preserving local texture and boundary detail information in cell images, the proposed method further strengthens the representation of structural correlations among different regions, and suppresses non-essential interferences such as background noise, staining variation, and imaging bias through stable discriminative feature modeling, thereby improving the model’s recognition capability for complex pathological cell images. Experimental results demonstrate that the proposed method achieves superior performance over competing methods on multiple evaluation metrics, including classification accuracy, precision, recall, and AUC. In addition, the effectiveness of each key module design and the rationality of the overall framework are further validated through ablation experiments, confusion matrix analysis, feature distribution visualization, and parameter sensitivity analysis.

Future research can further extend and deepen this work toward more complex pathological cytology scenarios. On the one hand, the proposed framework can be generalized to more types of brain tumor cells, multi-class pathological cell subtyping, and cross-center microscopic image analysis tasks, so as to verify its adaptability under broader medical data distributions. On the other hand, it is also possible to further incorporate cytological prior knowledge, region-level annotation information, and multimodal clinical data to construct an intelligent pathological analysis model with high accuracy, strong generalization ability, and improved interpretability, thereby providing more reliable technical support for medical-assisted diagnosis and intelligent cytology research.

## Data Availability

The original contributions presented in the study are included in the article/supplementary material, further inquiries can be directed to the corresponding authors.
